# Visualizing and trapping transient oligomers in amyloid assembly pathways

**DOI:** 10.1016/j.bpc.2020.106505

**Published:** 2021-01

**Authors:** Emma E. Cawood, Theodoros K. Karamanos, Andrew J. Wilson, Sheena E. Radford

**Affiliations:** a Astbury Centre for Structural Molecular Biology, School of Chemistry, University of Leeds, LS2 9JT, UK; b Astbury Centre for Structural Molecular Biology, School of Molecular and Cellular Biology, University of Leeds, LS2 9JT, UK; c Laboratory of Chemical Physics, National Institute of Diabetes and Digestive and Kidney Diseases, National Institutes of Health, Bethesda, MD 20892, USA

**Keywords:** Amyloid disease, Transient intermediate, Oligomer stabilization, Chemical tool, NMR, Single particle

## Abstract

Oligomers which form during amyloid fibril assembly are considered to be key contributors towards amyloid disease. However, understanding how such intermediates form, their structure, and mechanisms of toxicity presents significant challenges due to their transient and heterogeneous nature. Here, we discuss two different strategies for addressing these challenges: use of (1) methods capable of detecting lowly-populated species within complex mixtures, such as NMR, single particle methods (including fluorescence and force spectroscopy), and mass spectrometry; and (2) chemical and biological tools to bias the amyloid energy landscape towards specific oligomeric states. While the former methods are well suited to following the kinetics of amyloid assembly and obtaining low-resolution structural information, the latter are capable of producing oligomer samples for high-resolution structural studies and inferring structure-toxicity relationships. Together, these different approaches should enable a clearer picture to be gained of the nature and role of oligomeric intermediates in amyloid formation and disease.

## Highlights

• Methods to study structure, toxicity, and kinetics of transient amyloid oligomers.
• NMR and single particle methods can characterize lowly-populated oligomers.
• Chemical tools/antibodies stabilize oligomers for structural and toxicity studies
• A combination of methods is needed to fully characterize amyloid assembly pathways.

## Graphical abstract

**Figure:**
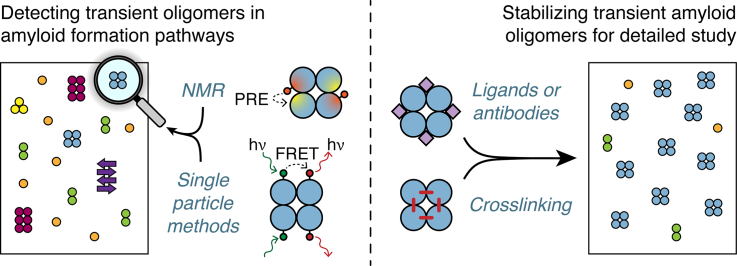

## 1 Introduction

**Fig. 1:**
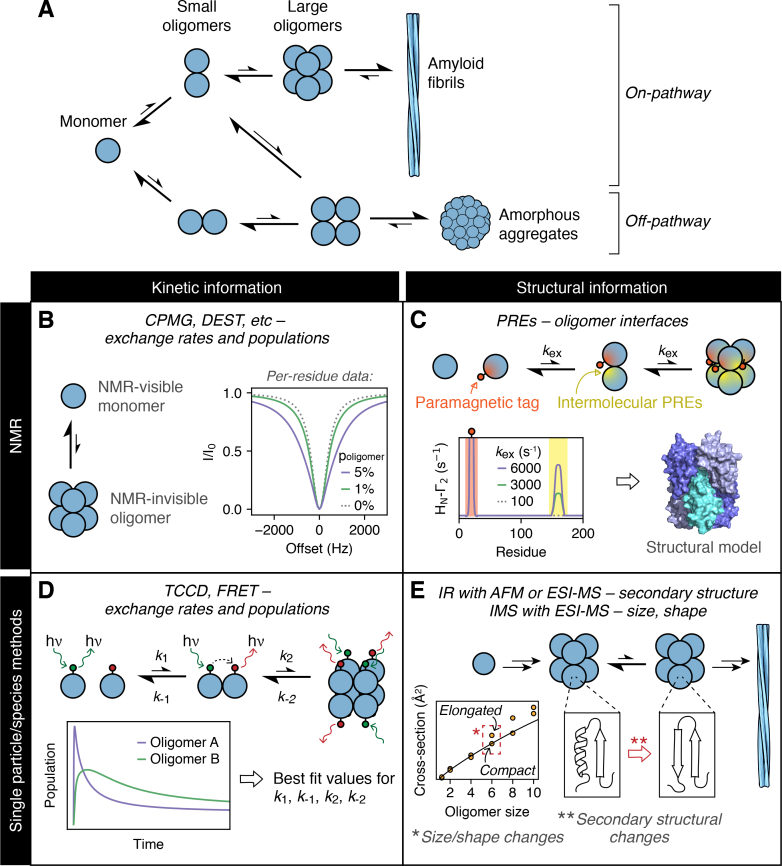
Understanding the complex mechanisms of amyloid fibril assembly using “non-perturbing” biophysical methods. A: A schematic of a generic protein self-assembly pathway. The self-association of particular proteins can lead to the formation of cross-β fibrillar structures (amyloid fibrils) but can also lead to the formation of aggregates which lie “off-pathway” from fibril assembly. Assembly intermediates are often lowly-populated, rapidly interconverting, and transiently formed, making them challenging targets for many biophysical techniques, with notable exceptions being NMR (B, C) [[31], [32], [33], [34], [35]] and methods which detect single particles or species (D, E) (e.g. single particle fluorescence - including FRET and 2-color incidence detection, TCCD - [36,37], SPFS [38], and ESI-MS [[39], [40], [41]]). Monomer-oligomer exchange rates and oligomer populations (p_oligomer_) can be obtained through the global fitting of oligomerization models to the observed NMR or single particle data (B, D). The structural information obtained through NMR and single particle/species methods is typically of low resolution but can provide insights into oligomerization interfaces (C), and structural transitions (E). The DEST and PRE data shown in B and C, respectively, were simulated by numerically solving the corresponding McConnell equations using a *B*_1_ field of 200 Hz, *R*_2_^B^ = 1000 s^−1^, *R*_2_^A^ = 10 s^−1^, and *R*_1_^A^ = 1.5 s^−1^. The PRE data shown in C were simulated with a distance between residue 160 and the MTSL label (on residue 20) of 1 Å in the excited state (5% population). The black line in the collisional cross-section plot in E represents the expected cross-sections for each oligomer size, assuming isotropic growth, and the dashed red box indicates the point in the self-assembly pathway at which the oligomers of this protein start to undergo structural transitions, as detected by IMS coupled to ESI-MS [17,41]. (For interpretation of the references to color in this figure legend, the reader is referred to the web version of this article.)

Amyloid fibril formation is a complex and multifaceted process, characterized by the self-assembly of proteins or peptides into insoluble cross-β deposits [1,2] (Fig. 1A). In healthy individuals, amyloid assembly pathways are highly regulated and accessible only to a small number of proteins, resulting in fibrils which can play important functional roles. These so-called “functional amyloid” fibrils can act as structural scaffolds, protein reservoirs, biofilm components, or be involved in the laying down and storage of long-term memories, amongst other biological roles [3,4]. However, normally soluble proteins and peptides can also be rendered amyloid-competent by specific mutations, changes to post-translational modifications, or changes to the intra- or extracellular conditions [1]. The aberrant and unregulated self-assembly events which subsequently occur are associated with an array of human disorders, ranging from Alzheimer's and Parkinson's diseases, to type II diabetes and dialysis-related amyloidosis [1,2].

For many disease-associated amyloid proteins, it has been recognized that soluble oligomers which form during, or as a result of, fibril assembly can be the major cytotoxic species associated with cellular dysfunction and disease onset [[5], [6], [7], [8], [9], [10]]. Consequently, there has been intense interest in the study of such assembly intermediates, with the aim to better understand the molecular mechanism(s) by which soluble oligomers form and transition into a cross-β structure, as well as to identify structural features associated with cytotoxicity [11]. Unfortunately, the complexity and heterogeneity of amyloid assembly pathways makes such analyses immensely challenging in vitro, let alone in vivo. Additionally, it is difficult to reconcile the vast range of oligomers observed by different research groups under varying experimental conditions. Oligomeric intermediates are typically metastable, lowly-populated, and interconverting with each other and fibril surfaces within the aggregating mixture [12,13]. As a consequence, the properties of specific oligomers cannot be readily determined in detail using most bulk solution methods, as such approaches typically do not allow information to be extracted about individual species within these diverse populations. The structural and kinetic study of oligomeric intermediates thus requires the use of techniques capable of dissecting the properties of individual species within complex mixtures, coupled with careful data analysis to extract the relevant information about their population, lifetime, and structural features. Some of the most powerful methods include those which can directly detect single particles (such as single particle fluorescence, e.g. fluorescence resonance energy transfer (FRET) [14,15], and single particle force spectroscopy (SPFS) [16]) or single species (such as electrospray ionization mass spectrometry (ESI-MS) [[17], [18], [19], [20]]). Solution-phase nuclear magnetic resonance (NMR) spectroscopy, which is unique amongst ensemble techniques for its ability to extract structural and kinetic information about different protein populations in atom-specific detail, can also be used for the characterization of lowly populated and rapidly interconverting species [21]. Additionally, molecular dynamics simulations using a variety of different force fields and approaches can provide insights into the potential repertoire of oligomers formed, which then require parallel experiments for their verification [[22], [23], [24], [25], [26]]. However, the aforementioned experimental approaches are not without their limitations – in particular, the structural resolution obtained using these techniques is generally insufficient to inform rational drug design approaches. Thus, there remains an on-going need to explore complementary methods for the characterization of amyloid oligomers.

The purposeful manipulation of an amyloidogenic system, with the aim of tuning the self-assembly landscape to favor specific events or species of interest, represents an alternative strategy for the study of amyloid self-assembly. Such an approach can reduce sample heterogeneity and, if used to bias the system towards oligomer formation, may allow mechanistic and structural information to be obtained about these assembly intermediates using a wider range of techniques than would otherwise be possible. Modulation of amyloid assembly can be achieved through the use of small molecule ligands, antibodies, or covalent protein modifications, to generate samples with a range of defined oligomer populations. In combination with methods such as NMR, kinetic modelling, and cell toxicity assays, these samples can provide insights into the role, disease-relevance, and structure of particular oligomeric populations, and validate potential routes for the treatment of amyloid diseases. There are now several examples where the stabilization (or destabilization) of specific species has reduced heterogeneity sufficiently to facilitate detailed structural studies using X-ray crystallography, thereby providing high-resolution insights into these potential therapeutic targets [[27], [28], [29], [30]].

This review provides an overview of the experimental methods and approaches which can be used to study the structure, kinetics, and disease-relevance of transient amyloid oligomers, and provides examples of their use in vitro. Although the ultimate goal of the amyloid field is to perform these experiments in vivo, in vitro studies allow us to explore the energy landscape of amyloid protein self-assembly and identify general trends between certain structural features (e.g. hydrophobicity, secondary structure) and toxicity. We discuss approaches through which observations can be made without intentional perturbation of the self-assembly process (e.g. using solution NMR, single particle methods, and native ESI-MS), as well as those through which the self-assembly equilibrium is deliberately modified to gain information about specific species. These “non-perturbing” and “perturbing” methods are complementary: most non-perturbing techniques are best suited to the determination of kinetic parameters (yielding information about rates of interconversion and population lifetimes), while the resolution of structural information they can obtain is typically lower. By contrast, perturbing methods offer the opportunity to analyze species using high-resolution structural methods, and hence enable detailed structure-toxicity relationships to be determined. The combination of both of these approaches provides an ideal experimental toolkit for generating more complete descriptions of the molecular mechanisms of amyloid fibril formation and their associated cellular toxicity.

## 2 Non-perturbing methods for the study of amyloid oligomers

### 2.1 Solution NMR

NMR spectroscopy has the capability to characterize transient and heterogeneous systems in all-atom detail, and thus has been widely utilized in the characterization of amyloid protein assembly [21,42,43]. Many powerful NMR methods are sensitive to the properties of “NMR-invisible” species (i.e. those <1% populated or of high molecular weight) that are in rapid exchange with the NMR-visible state(s) [21,44]. In the context of amyloid assembly, this means that the properties (e.g. size, timescale of formation) of lowly populated (ca. 0.5–15%) amyloid oligomers can be indirectly studied via the monomeric precursor, using experiments such as Carr-Purcell-Meiboom-Gill (CPMG) relaxation dispersion, chemical exchange saturation transfer (CEST), paramagnetic relaxation enhancement (PRE), life-time line broadening, dark exchange saturation transfer (DEST), and off-resonance *R*_1_ρ relaxation [42]. Using these methods, the relative populations and exchange rates (100–10,000 s^−1^) between species can be extracted, providing valuable information about the assembly process. Some experiments (e.g. CPMG relaxation dispersion and CEST) are designed to probe exchange with species whose relaxation properties are not very different to those of the monomer, and thus are best suited to the study of small oligomers [[45], [46], [47]]. By contrast, others, such as DEST, life-time line broadening, and off-resonance *R*_1_ρ [33], require exchange of the monomer with a higher molecular weight species, making them ideal for the study of large amyloid oligomers or the exchange between monomer and fibrils [32,48].

The application of these NMR methods, in combination with global fitting of data acquired at a range of protein concentrations and magnetic field strengths, can provide valuable insights into the mechanisms of self-assembly (Fig. 1B). Such an approach has recently been used to interrogate the early stages of aggregation of a minimal peptide from the protein huntingtin (associated with Huntington's disease), providing information about the populations, exchange rates, and secondary structure of oligomers formed by two competing aggregation pathways [31]. Similar strategies have been adopted for other amyloid proteins that form a range of oligomeric states and structures, revealing insights into the binding of amyloid β (Aβ) monomers to fibrils [49], the energy landscape of copper‑zinc superoxide dismutase (SOD1) aggregation [50], and the formation of β_2_-microglobulin (β_2_m) oligomers [35,51] – events which are associated with Alzheimer's disease, amyotrophic lateral sclerosis, and dialysis-related amyloidosis, respectively. However, as these methods rely on reversible chemical exchange, this places a limit on the protein concentrations which can be used. There is an apparent irreversibility of the amyloid pathway for samples prepared above their critical protein concentration, and so sample conditions must be carefully selected to minimize the formation of any highly aggregation-prone species, while maintaining a suitable signal-to-noise ratio.

An alternative NMR approach that can be used to study the assembly of amyloid oligomers involves tilting the energy landscape of aggregation in a controlled manner through the use of hydrostatic pressure [34]. Pressure allows the rapid disassembly of oligomeric species before they convert to pressure-resistant fibrils. This technique therefore permits study of the oligomerization equilibria that would not be accessible to the experiments described above under a constant atmospheric pressure. Pressure-jump NMR experiments, in which the pressure inside the NMR cell is rapidly altered (in a matter of milliseconds), have been used recently to study the interconversion of a highly unfolded Aβ monomer (at high pressure) with an oligomeric species (at low pressure), providing residue-specific information about oligomers that show the first signs of conversion into amyloid [34].

In addition to probing the kinetics of protein self-assembly, NMR can also be used to investigate the structural properties of amyloid oligomers [31,[52], [53], [54]]. NMR-derived structural insights into these protein complexes can be achieved through the determination of distance restraints from NMR experiments, which are then used to drive simulated annealing calculations [55] or other molecular dynamics approaches [56]. Due to the transient nature of the complexes present in the early stages of amyloid assembly, traditional nuclear Overhauser effect-based structural investigations typically fail and intermolecular distance restraints tend to instead be derived from PRE studies using paramagnetic spin labels (commonly MTSL; *S*-(1-oxyl-2,2,5,5-tetramethyl-2,5-dihydro-1*H*-pyrrol-3-yl)methyl methanesulfonothioate) [57] (Fig. 1C). When covalently attached at a specific site on a protein's surface, spin labels can be used to map oligomerization interfaces, even those that are short-lived (< 100 μs) [58]. Data from PRE experiments have been used to generate structural models of β_2_m dimers and hexamers [35,52] (Fig. 1C), tetramers formed by a minimal huntingtin construct [31], and homo-/heterodimers formed by α- and β-synuclein (the former variant being associated with Parkinson's disease) [53,54].

Due to the size limitations of solution NMR methods, it has remained challenging to investigate large oligomeric assemblies directly using solution NMR-based approaches. However, with the advance of ^13^C-methyl-TROSY methods as sensitive reporters of protein structure and dynamics, amyloid oligomers can be studied directly (provided that they exist in a sufficiently large population) [59]. Finally, the arsenal of magnetic resonance techniques to study amyloid formation would not be complete without mentioning solid-state NMR and electron paramagnetic resonance (EPR). Both techniques have contributed substantially to our understanding of the structures of amyloid fibrils [60,61], as well as oligomers of Aβ peptides [62,63] and α-synuclein [64], but are frequently geared towards stable, monodisperse samples (e.g. fibrils or stable oligomers), with some notable exceptions [65,66].

NMR currently remains the only ensemble method that can yield atom-specific structural and kinetic information (including rates of interconversion and lifetimes) for lowly populated, transient states, by observing their effect on the main species in solution (usually the monomer). However, rather than inferring such information from the interpretation of NMR data, it is also possible to directly observe transient, oligomeric species using single particle and other non-ensemble averaged methods, as described below.

### 2.2 Single particle and non-ensemble averaged methods

Single particle techniques are unparalleled in their ability to directly detect individual molecules and populations of interconverting species within heterogeneous mixtures. Single particle fluorescence methods (a term we use here to describe approaches which rely on fluorescent dyes, rather than the measurement of intrinsic protein fluorescence) and SPFS have both played key roles in the amyloid field, allowing kinetic and low-resolution structural information to be obtained for the species populated during fibril assembly [[67], [68], [69]]. Other non-ensemble averaged methods, such as ESI-MS, are also able to directly detect specific oligomer populations during assembly. When coupled to other techniques (e.g. ion mobility spectrometry, hydrogen-deuterium exchange, or infrared spectroscopy), ESI-MS is capable of providing structural information for these individual oligomeric species [18,19].

Single particle fluorescence experiments used for the study of amyloid assembly most commonly require preparation of dual-labelled protein samples, containing an equimolar mixture of protein molecules labelled with one of two distinct fluorescent dyes [67]. From such dual-labelled samples, oligomers can be detected by the observation of simultaneous bursts from the two fluorophores or, in cases where the emission of one fluorophore overlaps with the absorption of the other, by the ability of these molecules to undergo FRET (Fig. 1D) [10,36,37,[70], [71], [72], [73], [74], [75], [76], [77], [78], [79], [80]]. Different oligomer populations can be distinguished from one another based on the intensity of fluorescent bursts [79,80], the FRET efficiency [10,36,37], or the stability of the detected oligomers to particular experimental conditions (e.g. dilution into buffer with high or low ionic strengths, or the presence of proteases) [10,37,75,76,79,81]. By using such measurements to monitor the population of specific oligomers in an aggregating mixture over time, the resulting data can be fitted to self-assembly models to determine the rates of oligomer formation and interconversion, as well as thermodynamic parameters for these protein-protein interactions [36,37,76,78,82] (Fig. 1D).

While single particle fluorescence is immensely powerful, like any technique it relies on certain assumptions and has various limitations. The requirement to perform single particle fluorescence measurements under highly dilute solution conditions (several orders of magnitude less concentrated than the conditions ordinarily used to study protein aggregation in vitro) can limit the types of oligomers which can be observed. Typically, aliquots from a more concentrated aggregating mixture are taken and diluted immediately prior to single particle experiments, and thus unstable and/or highly transient oligomers may dissociate before the data are acquired [79]. However, this issue can be minimized through the use of microfluidic devices to speed up sample analysis [75,83] or by diluting aliquots into a solution of non-fluorescently labelled protein, rather than buffer alone [10]. An additional factor which must be considered is that most single particle fluorescence experiments make use of fluorescently-labelled protein samples, and therefore rely on the assumption that the incorporation of fluorescent dyes, which are often large and hydrophobic, does not perturb the assembly mechanism. This assumption may not always hold true [[84], [85], [86]], and the appropriate selection of fluorophores must be ensured by performing thorough control experiments. It is also possible to perform experiments with fluorescent probes which interact non-covalently with the protein of interest, and which can therefore be added after assembly has taken place [81,82,87,88].

SPFS relies on the use of mechanical force to perturb interatomic interactions and has been extensively used to study the formation of native protein contacts during folding [89]. However, non-native intermolecular contacts that take place during protein misfolding and self-assembly can also be studied through such methods. Atomic force microscopy (AFM) is the primary SPFS technique which has been used to study amyloid assembly, as the high forces accessible in this method are suitable for the study of amyloidogenic oligomerization events [16]; lower forces can be accessed using other, complementary SPFS methods [90], such as optical tweezers [[91], [92], [93], [94]]. Unlike single particle fluorescence measurements, where soluble oligomers formed at any stage during aggregation can be detected within a single experiment, AFM SPFS experiments tend to be designed to focus on specific oligomerization events – commonly dimerization. The lowering and raising of an AFM cantilever to and from a surface controls the formation and then drives dissociation of individual interactions [95]. For the study of dimerization events, one protein is typically attached to the cantilever tip, whilst the partner of interest (often the same protein) is immobilized on a surface on the sample stage [[96], [97], [98], [99], [100], [101], [102], [103], [104], [105], [106], [107], [108]]. Formation of higher oligomers can be studied using tandem repeat oligomers which are tethered between the surface and cantilever tip [109] or, more recently, using the flexible nanoarray (FNA) approach where a number of monomers are immobilized at various points along a flexible polymer [[110], [111], [112], [113]]. In both cases, the site of covalent attachment and the length/flexibility of the covalent linker need to be carefully selected to ensure that steric constraints do not alter the accessibility of key interaction interfaces or change the behavior of the protein. SPFS methods can yield information concerning the lifetime of specific protein-protein interactions and their strength (force resistance). Furthermore, the distance required to move the cantilever away from the surface to cause disruption of the oligomer can be used to infer the site of interaction [95,114].

In addition to its use as a SPFS method, AFM can be used as a surface-imaging technique to directly observe the formation and turnover of oligomers during amyloid assembly [68]. Recently, AFM has been coupled with infrared spectroscopy (AFM-IR) to image the secondary structure content of individual amyloid oligomers at the single aggregate level and to identify structural transitions which occur during the self-assembly of various amyloid proteins, including ataxin-3 (the causative agent of spinocerebellar ataxia type-3 or Machado–Joseph disease) [38], huntingtin exon 1 [115], and α-synuclein [116] (Fig. 1E). Such conformational conversion events are often rate-limiting steps in amyloid formation [117], and thus are key processes to characterize in structural and kinetic detail.

ESI-MS represents an alternative technique for the direct detection of individual monomeric conformers and oligomeric species formed during amyloid assembly. As a soft ionization method, ESI allows non-covalent interactions to be maintained when aggregating proteins are sprayed into a mass spectrometer from a volatile buffer solution [118]. ESI-MS has the advantage over the single particle methods discussed here in that no dye labels or immobilization strategies are required, with the mass accuracy of modern mass spectrometers making it straightforward to detect different oligomeric species that are co-populated [[17], [18], [19], [20]]. Most powerfully, ESI-MS can be directly coupled to other methods, such as ion mobility spectrometry (IMS; which allows oligomers with the same *m*/*z* ratio to be separated based on size and shape) [41,[119], [120], [121], [122], [123], [124], [125], [126], [127], [128], [129], [130], [131], [132], [133]], hydrogen-deuterium exchange and related covalent labelling experiments (revealing oligomer interfaces) [[134], [135], [136], [137], [138], [139], [140], [141], [142], [143], [144], [145], [146], [147]], and infrared spectroscopy (IR) [39,40] to yield structural information about the different species present in an aggregating mixture. These techniques have been used to detect structural transitions in amyloid assembly pathways [[39], [40], [41],^123^,^135^] (Fig. 1E), produce low-resolution structural models of specific intermediates [144,145,148], and infer the role of different oligomers in amyloid assembly and toxicity [120,130].

The primary limitation of methods which detect single particles or populations is their structural resolution. Unlike NMR, these techniques do not readily reveal residue- or atom-level information about amyloid assembly, and thus cannot facilitate structure-based ligand design approaches. We note, however, that the recent revolution in cryo-electron microscopy (cryo-EM), including advances in image processing algorithms, has allowed individual species within heterogeneous samples to be studied at high resolution [149]. While cryo-EM has already played a key role in the elucidation of fibril structures at near-atomic resolution [150], there are few examples of its application to amyloid oligomers [8]. This technique nonetheless has the potential to be used in the future to reveal high-resolution structural information for oligomeric samples at a “single species” level.

NMR and single particle methods remain invaluable for detecting individual oligomer populations and elucidating amyloid pathways. These approaches, in combination with methods which allow access to higher-resolution information (discussed below), facilitate progress towards more refined descriptions of amyloid assembly pathways.

## 3 Trapping transient oligomers to facilitate the characterization of amyloid self-assembly

Obtaining high-resolution structural and functional insights into specific amyloid oligomers typically requires the use of samples which predominantly contain a single species. While it is possible to reduce the heterogeneity of oligomer samples through size separation approaches [151], the degree of sample homogeneity achievable through this method is limited, as the self-assembly landscape may start to rapidly re-equilibrate after isolation of individual species. Careful control of sample preparation conditions (e.g. the availability of air-water interfaces, or buffer composition and pH) can bias self-assembly landscapes towards specific oligomeric states (Section 3.1), but the resulting oligomer distributions are often broad. Furthermore, such an approach does not address the experimental challenges which remain concerning how to trap and kinetically/structurally characterize amyloid intermediates in an in vivo setting. Four primary tools have emerged which can favor the production of narrow distributions of oligomers, or sometimes even a specific oligomer state, without requiring specific buffer conditions: oligomer-binding antibodies (Section 3.2), non-covalent small molecule ligands (Section 3.3), covalent ligands or protein modifications (Section 3.4), and crosslinking (Section 3.5).

### 3.1 Sample preparation strategies

For certain amyloid proteins, different sample preparation strategies have been established which have been shown to favor specific oligomer distributions. Lyophilization, followed by resuspension and incubation at high protein concentrations, has been used to promote the formation of kinetically-trapped α-synuclein oligomers that can then be further enriched by centrifugation, size-exclusion chromatography, or other size separation methods [152]. While oligomer distributions produced by this approach are still broad (predominantly 10-40mers, although species up to 90mers have been detected) [8,153,154], the enrichment of oligomers in these samples has nonetheless facilitated their study via a range of biophysical techniques [8,136,138,[153], [154], [155], [156], [157], [158]]. Notably, lyophilization has been used to produce α-synuclein samples for cryo-EM, leading to two low-resolution (18–19 Å) reconstructions of toxic cylindrical oligomers [8], and solid-state NMR, where structural properties of these same oligomers were compared with those of non-toxic, small molecule-stabilized oligomers [159] to understand the structural determinants of oligomer toxicity [64]. Similarly, samples prepared through incubation of amyloid proteins in carefully-selected buffers have allowed structure-toxicity relationships to be uncovered for oligomers formed by the N-terminal domain of the *Escherichia coli* HypF protein (prepared in additive-containing solutions) [160,161] and Aβ peptides (prepared in low salt buffers at low temperature [162,163] or in the presence of detergent micelles [26,164]), amongst others. In general, such studies have highlighted that structural features which promote promiscuous interactions with other cellular components (e.g. enhanced surface hydrophobicity or the presence of structured elements which can insert into membranes) are often associated with toxicity [26,64,165,166], while the proportion of β-sheet structure does not generally appear to play a clear role [167].

### 3.2 Using antibodies to probe the structure and toxicity of oligomers

An emerging therapeutic avenue for the treatment of amyloid diseases is the use of antibodies to capture and neutralize toxic oligomers, due to the ability of these proteins to bind target epitopes with high specificity and affinity [168,169]. Such molecular recognition properties also make antibodies ideal tools for stabilizing specific, transient amyloid intermediates for detailed study [170,171] and for detecting the presence of particular structural features [172]. The A11 antibody, in particular, has played an important role in the amyloid field [6]. Although A11 was raised against an oligomeric mimic of Aβ_40_ (in the form of gold nanoparticles coated with Aβ_40_ peptides), this polyclonal antibody recognizes pre-fibrillar, toxic oligomers formed by a range of amyloid proteins with diverse primary sequences and native folds, suggesting that toxicity is associated with a common set of structural features [6]. Understanding precisely what these structural features are, how much they vary between different amyloid proteins, and which oligomeric species within a given self-assembly pathway possess these characteristics is still not understood.

**Fig. 2:**
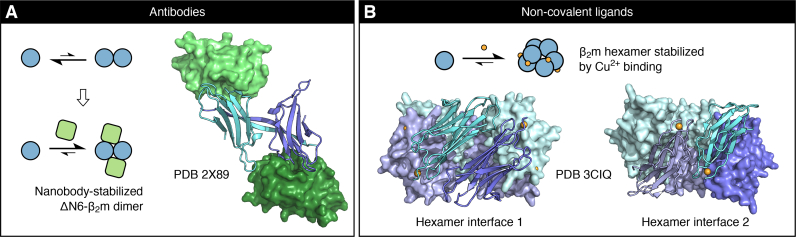
Non-covalent strategies for the stabilization of particular oligomer populations, exemplified by nanobody- and metal ion-stabilized β_2_m oligomers. A: Crystal structure of a dimer of a truncated β_2_m variant (ΔN6-β_2_m) stabilized by a nanobody (green) [28]. B: Crystal structure of a Cu^2+^-stabilized hexamer of the H13F β_2_m variant. The Cu^2+^ ions are colored in orange [29]. In both A and B, β_2_m protomers are shown in various shades of blue. (For interpretation of the references to color in this figure legend, the reader is referred to the web version of this article.)

In addition to antibodies which recognize specific structural features in a sequence-independent manner [6], antibodies (and antibody fragments) which bind to, and stabilize, specific oligomers formed by different amyloid proteins have also been developed: this includes antibodies which target oligomers of α-synuclein (SDS-stable dimers/tetramers [173] and SDS-stable trimers/hexamers [174]), Aβ (< 70 kDa oligomers [175,176] and larger aggregates [177]), and a truncated β_2_m variant (dimers [28]; Fig. 2A). Antibodies have also been developed that interfere with specific stages of amyloid assembly for particular proteins [178]. To guarantee specificity for amyloid oligomers (over monomers or fibrils), antibodies are often generated by screening against samples which have been enriched in oligomers [173,174,177], by screening against cyclic peptides which mimic predicted oligomer epitopes [175,176], by grafting amyloidogenic peptide fragments into the complementary-determining regions of canonical antibody scaffolds [179,180], or through rational in silico design methods [178,181]. The ability of antibodies generated using these approaches to bind oligomers in cell models or in ex vivo samples highlights the physiological- and/or disease-relevance of these antibody-stabilized structures, and in some cases has allowed the roles of specific oligomer sub-classes in disease to be interrogated [[174], [175], [176], [177], [178],^181^,^182^]. Oligomer-binding antibodies have also been used to facilitate structural studies of amyloid intermediates – this has been demonstrated by a nanobody-stabilized dimer formed by a truncated β_2_m variant (ΔN6-β_2_m; Fig. 2A) [28] and a tetrameric fusion between an antibody and an Aβ peptide fragment [183], both of which yielded high-resolution crystal structures. Thus, in contrast with other approaches discussed thus far in this review, antibody-oligomer complexes have the potential to provide high-resolution insights into the conformational changes which an amyloid protein undergoes during the initiation of self-assembly. Where it is possible to assess the toxicity of such stabilized oligomer complexes, structural features that are associated with disease can be inferred.

### 3.3 Stabilizing oligomers using non-covalent small molecules

Non-covalent small molecules offer an alternative approach to capture amyloidogenic intermediates. In a similar manner to antibodies [178], such ligands can be invaluable tools to interfere with distinct microscopic events in amyloid self-assembly [[184], [185], [186], [187]], although identification of such compounds is complicated by the poor ligandability of most monomeric amyloid precursors (which are commonly intrinsically disordered or partially folded). The discovery of non-covalent ligands specifically for the oligomeric forms of amyloid proteins presents additional challenges, as protein-protein interactions are inherently difficult to modulate with small molecules [188]. While small molecules have been identified that stabilize natively oligomeric amyloid precursors – e.g. the functional tetramers of transthyretin (the aggregation of which results in familial amyloid polyneuropathy) and immunoglobulin light chain dimers (involved in light chain amyloidosis) [[189], [190], [191], [192]] – the identification of compounds that bind specifically to non-native, oligomeric assembly intermediates is far more challenging: less structural information is available for non-native intermediates to guide ligand design and it can be difficult to detect oligomer-specific interactions due to the low population of these species. Screening methods [193] that are well-suited to the identification of oligomer-binding compounds include kinetics approaches, which can identify the microscopic events that are modulated by a given compound [187], and ESI-MS (sometimes also coupled with IMS), which is sufficiently sensitive to detect lowly-populated intermediates and can identify the oligomeric state of the ligand-bound species [194].

In addition to compounds which are identified through screening, small molecules and other ligands which are thought to modulate amyloid assembly in vivo can be used in vitro to gain insight into assembly pathways. In particular, several metal ions with known or proposed roles in amyloid disease have been shown to promote the formation of amyloid oligomers [[195], [196], [197]]. A striking example of this was demonstrated by Calabrese et al., where Cu^2+^-mediated stabilization of a β_2_m variant hexamer allowed a high-resolution structure to be obtained of this oligomer by X-ray crystallography, showing a ring-like arrangement of protomers with a native-like fold (Fig. 2B) [29].

For natively disordered amyloid proteins, obtaining discrete oligomer populations through non-covalent binding is immensely challenging, as small molecule or metal ion binding tends to result in a distribution of oligomers [159,[198], [199], [200], [201], [202], [203]]. Nonetheless, through bulk structural measurements in combination with assays in cells, structure-toxicity relationships have been obtained for various natively disordered amyloid proteins, including α-synuclein [159,202,203] and Aβ peptides [159,200,201,204,205].

### 3.4 Covalent ligands and protein functionalization

In recent years, covalent modification has become an increasingly utilized tool for the modulation of protein-protein interactions [[206], [207], [208], [209], [210], [211], [212], [213], [214], [215]]. A covalently bound small molecule may exert an effect on a protein or protein complex either via (a) non-covalent interactions (which have been reinforced by the covalent bond), or (b) by modifying the protein's surface properties or topography. We will refer to these two categories of modifications as “covalent ligands” and “protein functionalization”, respectively. Covalent ligands can offer improved affinity and selectivity over their non-covalent analogues [213,216,217], thereby overcoming some of the difficulties associated with using small molecules to target the dynamic and poorly structured oligomers formed by many amyloid proteins. Modification of a protein's behavior through functionalization does not require the conjugated chemical moiety to have a high non-covalent affinity for the target; it can instead effectively act as a chemical post-translational modification [218,219], altering the protein's surface properties [27,220] or acting as a steric block [30]. We also note that sequence variation can be considered as a special case of protein functionalization which alters the chemistry and/or steric bulk of protein sidechains without the need for chemical modifications to be performed post-translationally. The effect of sequence variation on the thermodynamic stability of amyloid precursors and oligomers, and on the kinetics of protein self-assembly has been extensively studied and reviewed elsewhere [29,[221], [222], [223], [224], [225], [226], [227], [228]].

**Fig. 3:**
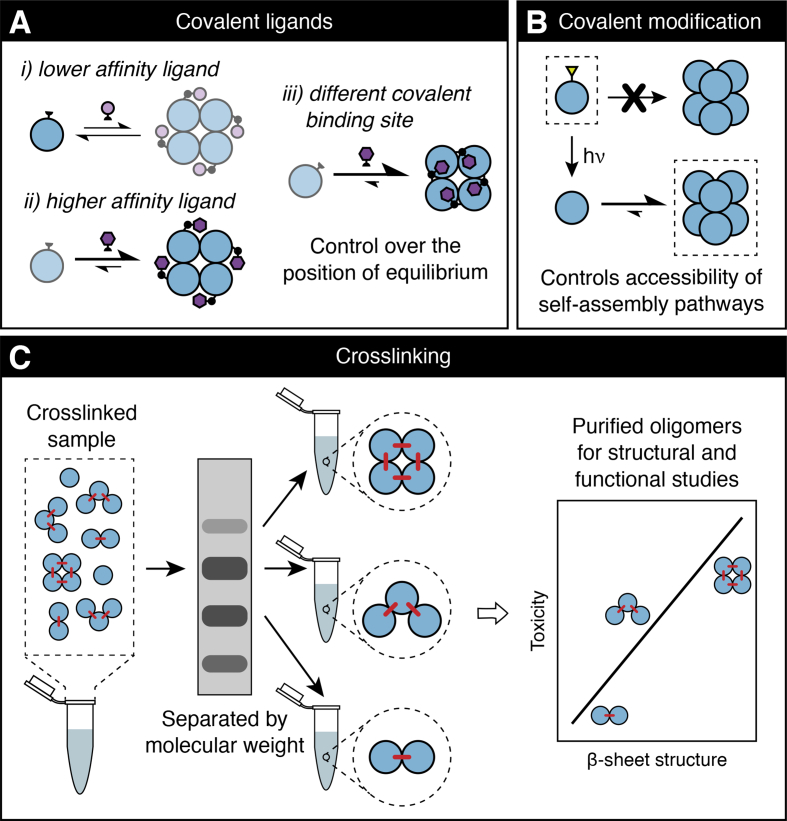
Covalent strategies for the control of oligomer populations in amyloid protein samples. A: Covalent ligands have been shown to be powerful modulators of oligomerization equilibria and can allow oligomer distributions to be tuned based on the affinity and location of the protein-ligand interaction [27]. B: The use of photolabile groups (e.g. *N*-2-nitrobenzyl) to sterically or chemically prevent amyloid proteins and peptides from accessing particular regions of the self-assembly landscape can allow oligomer populations to be controlled in a UV-dependent manner [30]. C: Covalent crosslinking using the PICUP or diazirine-based approaches represents a means of trapping a distribution of oligomers formed by a self-assembling protein, and the resulting crosslinked species can be separated (e.g. by SDS-PAGE, or using other, higher-throughput size/mass separation approaches) for individual structure and toxicity studies [[239], [240], [241]].

Both categories of covalent modification have shown great promise in dissecting the structure and toxicity of discrete oligomeric species in amyloid assembly, as has recently been demonstrated by a panel of small molecules used to stabilize a tetramer formed by the ΔN6 variant of β_2_m [27]. These compounds, which were identified through disulfide tethering [229,230], were found to be capable of modulating ΔN6-β_2_m oligomer populations, primarily by acting as covalent ligands. A series of samples was produced where the tetramer population could be varied (from ca. 5–95%) based on the site of covalent modification and the identity of the small molecule. These samples were used to determine a high-resolution structure of ΔN6-β_2_m tetramers and demonstrate their role in inhibiting amyloid fibril formation, through the use of X-ray crystallography, solution NMR, and functional assays (Fig. 3A) [27]. The ability of such covalent small molecules to generate specific oligomer populations in a controlled manner makes this a promising platform to study oligomer structures and structure-toxicity relationships for other amyloid proteins.

While the strategies discussed above have focused on methods to stabilize particular oligomeric states, sample homogeneity can also be improved by destabilizing or preventing the formation of other species in an amyloid assembly pathway. For example, the formation of inter-peptide backbone hydrogen bonds is essential for fibril formation [231] and so blocking the ability of the backbone to form these non-covalent interactions provides a means to prevent a protein from accessing certain regions of the self-assembly energy landscape. Chemical or steric blocking of backbone hydrogen bonds can be achieved through the incorporation of certain backbone mimics, such as the tripeptide β-strand mimic “Hao” [232] or through amide *N*-alkylation [233]. These approaches have been shown to restrict the self-assembly of a range of model amyloid peptides, including those derived from Aβ [30,[234], [235], [236]], tau (involved in Alzheimer's disease and other neurodegenerative disorders) [234], β_2_m [237], and islet amyloid polypeptide (IAPP; associated with type II diabetes) [238]. Recently, it has also been demonstrated that such covalent modifications can be applied in a reversible manner to tune oligomer populations with temporal control. The use of photolabile *N*-2-nitrobenzyl groups in place of (or in combination with) *N*-alkylation, for example, can allow precise control over amyloid oligomer populations, and hence cytotoxicity, of model Aβ peptides (Fig. 3B) [30]. The application of this approach to other model peptides and full-length amyloid proteins could thus drive novel insights into the role of different assembly intermediates and structural features in amyloid disease.

### 3.5 Crosslinking strategies

Crosslinking reactions which are rapid and indiscriminate in their amino acid preferences offer promising opportunities for capturing snapshots of transient oligomers formed by amyloidogenic proteins or peptides [242]. Such reaction characteristics are partially or fully exhibited by both the photo-induced crosslinking of unmodified proteins (PICUP) approach [243] and diazirine-based photochemical crosslinking [244]. The enhancement of oligomer stability resulting from covalent crosslinking renders the assembly intermediates within such samples amenable to purification by size separation approaches, allowing pure oligomer samples to be prepared for detailed structural and functional analysis [239,240,245] (Fig. 3C).

PICUP is a rapid, radical-based crosslinking method which can be used without the need for prior functionalization of the target protein with photoreactive groups [246]. Instead, metal complexes (typically tris-bipyridyl ruthenium(II)) are oxidized in the presence of visible light and subsequently abstract single electrons from amino acid sidechains (commonly tyrosine, cysteine, tryptophan, and methionine), rendering them capable of forming covalent bonds with suitable proximal residues [247]. PICUP has become an established method for the stabilization and study of amyloid oligomers [240,[248], [249], [250]], and the purification of crosslinked samples to yield individual oligomers has been demonstrated using a sodium dodecyl sulfate polyacrylamide gel electrophoresis (SDS-PAGE) extraction method [239,240] (Fig. 3C). The ability to obtain individual samples of well-defined (by mass, if not conformation), stabilized oligomers through PICUP has allowed the structure and toxicity of different Aβ oligomers to be probed. Ono et al. successfully isolated crosslinked Aβ_40_ dimers, trimers, and tetramers (all with at least 94% purity) and were able to individually characterize the secondary structure, amyloidogenicity, and cytotoxicity of each species, ultimately finding a correlation between all these variables [239]. With some modifications [251], the PICUP crosslinking approach, with subsequent oligomer purification, has also been applied to Aβ_42_ [240].

Despite the advantages of PICUP crosslinking chemistry, the requirement for an amino acid sidechain radical to encounter a radical scavenger or a readily oxidizable sidechain means that intermolecular crosslinking between protein molecules can occur through diffusion-controlled collision events, independently of genuine protein-protein interactions [248]. Such events can be avoided through the use of diazirine-based crosslinking, as the carbenes which form upon irradiation of diazirine groups with UV light have nanosecond lifetimes and are rapidly quenched by reaction with solvent if no crosslinkable residues are in proximity [252]. Although diazirine groups have to be introduced into the target protein or peptide by synthesis [25,241,253], covalent modification (e.g. via cysteine using methanethiosulfonate-functionalized diazirines [254]), or the use of unnatural amino acids during protein expression [255,256], the advantages offered by the short lifetime of the carbene, the high yield of potential crosslinks formed, and the rapidity of their formation when using LED illumination (reducing irradiation times from minutes or hours to seconds) [254] make diazirines attractive tools for capturing transient amyloidogenic interactions [25,241,244,253].

In theory, protein photo-crosslinking is well-suited to trap disease-relevant oligomers in cells or in vivo, as well as to identify the cellular components with which these oligomers interact. However, in practice, such an approach presents many challenges, notably the potential for very low signal-to-noise ratios due to the vast number of species within cells to which an amyloid protein can crosslink, as well as the complexities associated with subsequent data analysis. The use of crosslinking reagents with extremely short lifetimes and which offer the capacity for precise temporal control, such as diazirines, in combination with a method for enrichment of the crosslinked species (e.g. through affinity tags or alkyne-functionalized crosslinkers) addresses some of these challenges. Such an approach, in combination with quantitative proteomics has already been shown to be capable of identifying low-affinity, non-amyloidogenic protein-protein interactions in living cells [257]. Photo-crosslinking thus holds great potential for the study of amyloid assembly in physiologically- and disease-relevant systems.

When attempting oligomer-trapping methods, and particularly crosslinking strategies, it is important to keep in mind that while oligomer stabilization can dramatically improve sample homogeneity and offers advantages for structural characterization, the metastable and dynamic nature of amyloid intermediates can also be an important characteristic, particularly when considering oligomer toxicity. Oligomer dynamics, including protomer dissociation and exchange, can play important roles in toxicity [64,258]. While some crosslinked oligomers have been observed to undergo detectable dynamic motions [250], in other cases, crosslinking has been observed to suppress dissociation events which would otherwise contribute towards cell death [259]. It is therefore vital to employ a range of approaches to unpick the nature and potential cytotoxicity of different oligomers during amyloid assembly, keeping in mind that methods which are best suited to generating samples for high-resolution structure elucidation and those best suited for assessing oligomer toxicity are not necessarily the same.

## 4 Conclusion

Amyloid assembly intermediates are intimately involved in amyloid diseases, often representing key cytotoxic species which interact aberrantly with each other and other cellular components [11]. While the challenges presented by the transient nature, dynamics, and heterogeneity of these oligomeric intermediates have hindered structural and functional studies, continual advances in biophysical methods and chemical tools have allowed increasingly detailed insights to be gained. In this review, we have explored the role that NMR and single particle/species methods can play in gaining kinetic and low-resolution structural descriptions of lowly populated and transient oligomeric intermediates. In addition, strategies which allow specific oligomer populations to be stabilized (e.g. through the use of antibodies, small molecule ligands, or chemical crosslinking) can facilitate higher-resolution structural studies and investigation of detailed structure-toxicity relationships. It is important, however, to keep a balance between stabilizing a sample sufficiently to make it amenable to analysis through the desired methods and avoiding tipping the oligomerization equilibria to biologically- or disease-irrelevant species. The different strengths and caveats of the aforementioned methods make all these techniques complementary and emphasize the need to employ a wide range of integrated methods and tools from chemistry, biophysics, structural biology, and cellular biology to gain a complete description of these disease-relevant self-assembly pathways and to inform the rational design of much-needed treatments.

## Declaration of competing interest

The authors declare that they have no known competing financial interests or personal relationships that could have appeared to influence the work reported in this paper.
